# Ecophysiology of Endangered Plant Species *Saussurea esthonica*: Effect of Mineral Nutrient Availability and Soil Moisture

**DOI:** 10.3390/plants12040888

**Published:** 2023-02-16

**Authors:** Agnese Gailite, Una Andersone-Ozola, Ineta Samsone, Andis Karlsons, Gederts Ievinsh

**Affiliations:** 1 Latvian State Forest Research Institute “Silava”, 111 Rigas Str., LV-2169 Salaspils, Latvia; 2 Department of Plant Physiology, Faculty of Biology, University of Latvia, 1 Jelgavas Str., LV-1004 Rīga, Latvia; 3 Institute of Biology, University of Latvia, 4 Ojāra Vācieša Str., LV-1004 Rīga, Latvia

**Keywords:** chlorophyll, chlorophyll *a* fluorescence, ecophysiology, mineral nutrient availability, rare and endangered plants, soil moisture

## Abstract

*Saussurea esthonica* is an endangered plant species typical for wet inland habitats such as calcareous fens. Due to its limited population size and distribution, non-invasive sampling of is important in the research of *S. esthonica*. The aim of the present study was to assess the effect of mineral nutrient availability and substrate moisture on the growth, physiological status, and mineral nutrition of *S. esthonica*. The non-destructive measurement of physiological parameters was performed in native habitats during three vegetative seasons, followed by two experiments in controlled conditions. Soil at the two Estonian sites had a relatively larger similarity in the composition of plant-available mineral nutrients in comparison to the two Latvian sites. The chlorophyll *a* fluorescence parameter Performance Index correlated with the total precipitation in the respective month before measurement, but no significant relationship with other environmental variables was found. For mineral nutrient experiments, plants were grown in four substrates with different mineral nutrient composition, resembling that of soil at different *S. esthonica* sites. Plant growth and physiological indices were significantly affected by the mineral composition of the substrate. Differences in leaf and root mineral nutrient concentrations of *S. esthonica* plants in part reflected differences in substrate mineral concentration. To evaluate the effect of soil moisture on growth and photosynthesis-associated parameters of *S. esthonica*, plants were cultivated in “Pope+” substrate at four different moisture treatments (dry, normal, wet, and waterlodged). The most intense growth of *S. esthonica* plants was evident in waterlodged conditions, which decreased with a decrease in soil moisture. The biomass of leaves increased by 106% and that of the roots increased by 72% as soil moisture increased from dry to normal. For waterlodged plants, leaf biomass increased by 263% and root biomass increased by 566%, in comparison to that for plants cultivated in dry substrate. Substrate drying had a more negative effect on the growth of *S. esthonica* plants in comparison to that of waterlodging, and this can be directly linked to prevalent hydrological conditions of an alkaline fen habitat native to the species. Therefore, the preservation of the natural water regime in natural habitats is critical to the conservation of the species.

## 1. Introduction

The development of conservation strategies of endangered plant species depends on a comprehensive knowledge of the biology of particular plants, including biogeography, population dynamics, genetic diversity, reproduction potential, physiological adaptations, etc. In this regard, ecological and genetical aspects of the conservation of rare and endangered plant species have mostly been considered [1]. However, there is a growing awareness that knowledge of the ecophysiology of these species is critically important for their conservation, especially in the context of global climate change [2,3]. This has been reflected in part by an increasing number of publications in the field as well as appeals for the need for mutualistic interactions between ecophysiology and different ‘omics’ technologies [4]. Physiological ecology is critical for understanding environmental conditions affecting the performance and distribution of the species as many threats affecting the persistence of endangered species may have physiological causes [5,6,7,8,9]. Physiological experiments are necessary components of the integrative approach in adaptation studies for understanding the effects of genetic variation on phenotype, performance, and fitness [10].

*Saussurea esthonica* Baer ex Rupr. (syn. *Saussurea alpina* subsp. *esthonica* (Rupr.) Kupffer) is a perennial species of the Compositae family. The genus *Saussurea* consists of about 400 species with a center of origin in Central and Eastern Asia [11]. On the territory of the European Union, *Saussurea esthonica* grows only in a limited number of sites in Estonia and Latvia [12]. While there is an ongoing scientific debate on the taxonomic affiliation of *S. esthonica* either as a species or subspecies of *Saussurea alpina* [12,13], it is well established that both taxa have very different habitat preferences. *S. esthonica* characteristically grows in wet inland habitats: spring fens, rich paludified grasslands, and *Molinia* grasslands; while *S. alpina* is a typical arcto-alpine species [12].

Factors that affect the distribution and physiological performance of *S. esthonica* are not well understood. Specific edaphic conditions could be one of the determining factors, as natural wetland soils are characterized by a relatively high heterogeneity with respect to their essential mineral nutrient concentrations [14]. In Latvia, *S. esthonica* is found only in the European Union protected habitat “7230 Alkaline fens” [15]. This *S. esthonica* habitat is associated with relatively calcareous, alkaline soils, rich in Ca, Mg, and K, but poor in N and P. Another factor faced by *S. esthonica* in natural sites could be the variation in the soil water level with relatively frequent episodes of soil waterlodging.

Given the conservation status of endangered plant species, especially those protected by legislation, as in the case of *S. esthonica* in Latvia, opportunities for ecophysiological studies with invasive sampling are very limited. For this reason, the only way to obtain information about the physiological state of plants in natural habitats is through non-destructive analytical methods such as chlorophyll content analysis and chlorophyll *a* fluorescence measurements. Chlorophyll *a* fluorescence measurement allows the non-destructive assessment of different functional and adaptation-oriented aspects of the photochemistry of photosynthesis, and it is often used for studies with rare and endangered plant species [7,8,16]. Most importantly, successful attempts have been made to correlate various fluorescence-derived aspects of the photochemistry of photosynthesis with the physiological performance of rare plant species in native conditions [6,17,18,19,20]. A comparison of the physiological fitness of individuals from different populations by means of chlorophyll *a* fluorescence analysis may provide valuable information on the putative viability of respective populations useful for conservation purposes.

Different biotechnological approaches offer additional opportunities for plant conservation research and associated practical applications [21,22]. Thus, tissue culture has been successfully used both for conservation needs and the propagation of plant material of rare and endangered plants species [22,23,24]. Seed-derived tissue culture as a means of the propagation of rare and endangered plant species for conservation and research is extremely useful. In particular, tissue culture of *S. esthonica* was successfully initiated with explants obtained from germinated seedlings and a relatively high rate of multiplication was achieved [25].

As an implementation of a nature-saving approach, the aim of the present study was to find environmental factors affecting the physiological performance of *S. esthonica* using a non-destructive experimental strategy involving field study based on soil analysis and physiological measurements of performance, and experiments in controlled conditions using plant material propagated by tissue culture. Two hypotheses were tested: (i) that *S. esthonica* plants are well-adapted to a relatively wide range of soil mineral nutrient concentrations, and (ii) that *S. esthonica* plants can tolerate high soil moisture being relatively susceptible to drought.

## 2. Materials and Methods

### 2.1. Field Study

**Figure 1:**
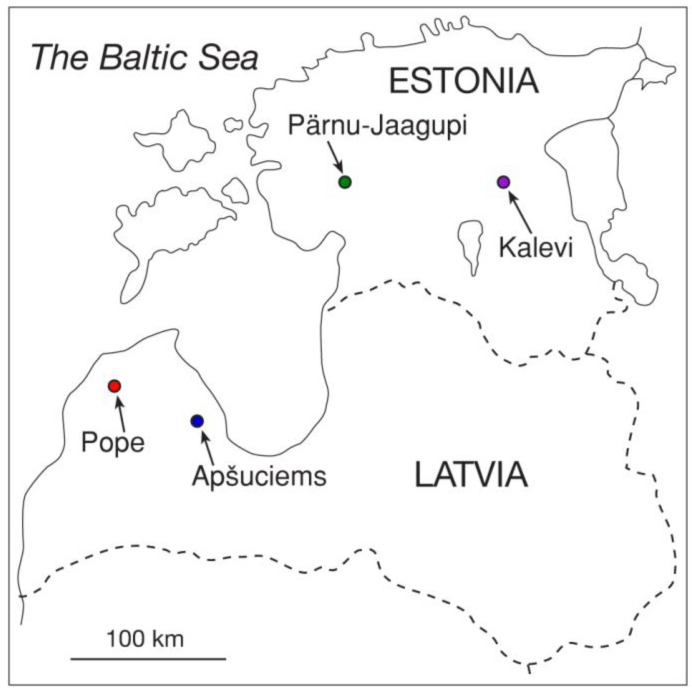
Location of study sites of *Saussurea esthonica* in Latvia and Estonia.

The sites with *S. esthonica* in Latvia are protected by legislation as a special area of conservation and Natura 2000 sites: nature reserve “Popes zāļu purvs” near Pope (LV0830400; 21°51′28″ E, 57°22′26″ N) and micro-reserve “Dubļukrogs” near Apšuciems (LV0531900; 23°18′43″ E, 57°02′38″ N) (Figure 1 and Figure S1). Both are calcareous fens with other protected plant species including *Carex davalliana, Pinguicula vulgaris, Dactylorhiza fusci, Dactylorhiza incarnata, Primula farinosa, Platanthera bifolia* (Pope), and *Schoenus ferrugineus, Dactylorhiza incarnata, Gymnadenia conopsea,* and *Primula farinosa* (Apšuciems; data from http://eunis.eea.europa.eu, accessed on 15 May 2012). Two *S. esthonica* sites chosen for analysis in Estonia represent wooded meadow near Pärnu-Jaagupi (24°30′4″ E, 58°37′49″ N) and calcareous fen near Kalevi (26°26′19″ E, 58°42′24″ N) (Figure S1). Climate conditions in Latvia and Estonia are largely determined by the location in the temperate climate zone on the coast of the Baltic Sea and the Gulf of Riga. There are well-defined signs of a maritime climate: a small amplitude of average temperatures in January and July, increased precipitation, and unstable weather conditions. The average annual air temperature is 6.8 °C, the amount of annual precipitation is 686 mm.

Non-destructive measurements of chlorophyll *a* fluorescence and chlorophyll content were performed in natural sites of *S. esthonica* for three years (2009–2011), three times within the vegetation season (from June to September) in both Latvian *S. esthonica* populations and once a year (in July) in both populations in Estonia. The number of flowering individuals in different years in the study sites was highly variable, in an extreme case, only 19 [26]. At least eight flowering and seven vegetative plants were analyzed at each site at every time point. Flowering plants were designated as individuals that formed a stalk and developed flowers within the particular season. Vegetative individuals remained in the rosette state.

Chlorophyll content was measured by a chlorophyll meter SPAD 502 (Konica-Minolta, Tokyo, Japan). For each plant, all leaves of appropriate size were used for measurement, with five to ten separate chlorophyll content readings per leaf. For each leaf, an average value was calculated. Chlorophyll *a* fluorescence parameters were measured using a chlorophyll fluorometer Handy PEA (Hansatech Instruments, Pentney, King’s Lynn, UK). Three to five leaves per plant were darkened with plastic clips and fast chlorophyll fluorescence induction kinetics were measured after 20 min as described further.

In July of 2011, soil samples were collected in all four sites. Three representative samples were taken at each site and analyzed separately for plant-available soil mineral nutrient concentrations as described further. Soil samples were collected in the plant root zone, and five individual samples were taken, which were then mixed to form one representative sample.

To analyze a possible effect of air temperature and precipitation on physiological parameters of *S. esthonica*, meteorological data from nearby meteorological stations were used (http://www.meteo.lv (accessed on 15 May 2012) and http://rp5.ru (accessed on 15 May 2012)).

### 2.2. Effect of Mineral Nutrition in Controlled Conditions

In general, treatments used in the mineral nutrient experiment mimicked soil at both Latvian sites as well as soil at Pope enriched in some minerals (Table 1). As a control, commercial peat substrate KKS-1 (Laflora, Dobeļi, Latvia) with the addition of mineral nutrients in concentrations optimal for a majority of cultivated plant species [27] was used. As an exception, dolomite dust was added to all substrates to reach the same calcium level as for native soils. The second mix was based on peat substrate KKS-M3 (Laflora, Dobeļi, Latvia) which contained mineral nutrients in concentrations similar to those in the Apšuciems site. The third substrate was based on KKS-M3 and was similar to the soil from the Pope site. In order to check if increased nitrogen and sulfur in this soil mix could lead to improved growth and physiological performance of *S. esthonica* plants, the fourth treatment was based on the Pope substrate with the addition of nitrogen and sulfur at the optimal concentrations and designated as “Pope+”.

Plant material for the experiment was propagated using tissue culture as described previously [25]. Ex vitro acclimated plants at the three-leaf stage were individually planted in plastic containers (9 × 9 × 10 cm) containing appropriate substrates and allowed to acclimate for three weeks. Ten plants per treatment were used. Plants were cultivated in a growth chamber at 23/20 °C day/night temperature, and photosynthetically-active radiation was provided for 16 h with a photon flux density of 100 µmol s^–1^ m^–2^. Plants were watered with deionized water when necessary to maintain a stable mass of containers ±10%. The experiment lasted for 16 weeks. Leaf number and plant vitality was estimated throughout the experiment by visually scoring every individual plant 0 to 5 points (0, no above-ground parts visible; 5, excellent). Leaf chlorophyll analysis by a chlorophyll meter and chlorophyll fluorescence measurements were performed once a week using five plants per treatment. For chlorophyll analysis, all leaves of appropriate size were used with five to ten independent measurements per leaf. Chlorophyll *a* fluorescence was measured in three to five leaves per plant darkened for at least 20 min before analysis using plastic leaf clips.

At the end of the experiment, plants were harvested, separated into roots and leaves, and samples for tissue nutrient analysis were collected.

### 2.3. Effect of Substrate Moisture in Controlled Conditions

Ex vitro acclimated plants at the three-leaf stage propagated by tissues culture were individually planted in plastic containers (9 × 9 × 10 cm) containing “Pope+” substrate (Table 1). Plants were cultivated in the same conditions as for the previous experiment. After three weeks of acclimation, plants were randomly assigned to four treatments differing in soil moisture. Five plants per treatment were used. According to the preliminary experiments, a “normal” regime was designated as 100 ± 5%, the “moderately dry” treatment contained 80 ± 5% of water by mass, the “moderately wet” treatment contained 150 ± 5% of water, the “waterlodged” treatment contained approximately 200% of water. The waterlodged conditions were maintained by placing growth containers in trays containing water at the 3 cm level. Individual containers were weighed twice a week and necessary water mass differences were maintained throughout the experiment using deionized water.

The experiment lasted for 13 weeks. Leaf number and plant vitality were estimated throughout the experiment. Once a week, leaf chlorophyll content and chlorophyll *a* fluorescence were measured as described for the previous experiment. At the end of the experiment, plants were harvested, separated into roots and leaves, and samples for tissue nutrient analysis were collected. Used substrates were also analyzed for mineral nutrient content.

### 2.4. Analysis of Mineral Nutrients

Soil samples were air dried and sieved through a metal sieve (2 mm mesh size). Soil samples were extracted with 1M HCl in a 1:5 soil to extractant volume ratio. Plant samples were dry ashed in concentrated HNO_3_ vapors and re-dissolved in 3% HCl. Nutrient concentrations were measured in respective extracts by means of atomic absorption spectrophotometry (Ca, Mg, Fe, Cu, Zn, Mn), colorimetry (N, P, Mo, B), and flame photometry (K, Na) as described previously [28].

### 2.5. Data Analysis

Results were analyzed by KaleidaGraph (v. 5.0, Synergy Software, Reading, PA, USA). Statistical significance of differences between all sites was evaluated by one-way ANOVA followed by post-hoc analysis (Tukey’s HSD). Heat map generation and cluster analysis were performed by the freely available web program ClustVis (http://biit.cs.ut.ee/clustvis/, accessed on 2 October 2023) [29]. Hierarchical clusters were generated by the average linkage method with correlation distance.

## 3. Results

### 3.1. Field Study

The aim of the field experiment was to find putative site-specific differences in the physiological performance of *S. esthonica* within three vegetation seasons and to compare them with soil characteristics, air temperature, and precipitation data.

Soil samples in *S. esthonica* sites were characterized by a relatively good N availability (except for Pope and Kalevi), low P content (except for Pärnu-Jaagupi), low K content, and high Ca concentration in a decreasing sequence: Apšuciems > Pope > Kalevi > Pärnu-Jaagupi) (Table 2). Considerable variability was found for soil Mg content, decreasing in a sequence: Pärnu-Jaagupi (high) > Kalevi (high) > Apšuciems (low) > Pope (slightly deficient). While the S level was deficient in soils at Pärnu-Jaagupi, Kalevi, and Pope, an extremely high S content was found in soil at Apšuciems. In contrast, Fe was at a high level at all sites except Pope, where it was deficient. Mn content was extremely high (near-toxic levels) in Apšuciems, excessively high in Pärnu-Jaagupi, while it was near to optimum in Pope and Kalevi. The level of Zn and Mo was close to optimum at all sites, while B was extremely high in Apšuciems and high in both Pope and Kalevi. Cu content was slightly deficient in all sites except Pärnu-Jaagupi. 

**Figure 2:**
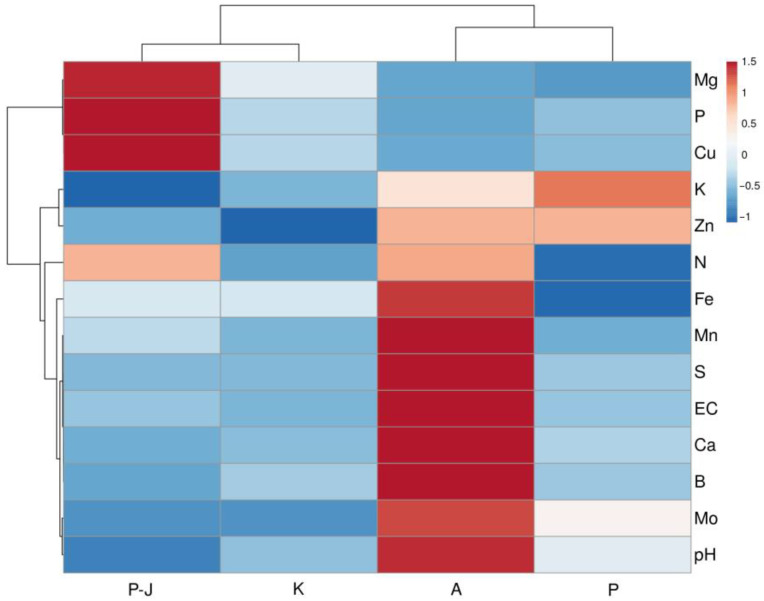
Heatmap and cluster analysis of soil characteristics in study sites of *Saussurea esthonica*. EC, electrical conductivity; P-J, Parnu-Jaagupi; K, Kalevi; A, Apšuciems; P, Pope.

While plant-available soil mineral nutrient profiles in different *S. esthonica* sites showed significant differences (Table 2), soil at the two Estonian sites had a relatively larger similarity, in comparison to the other sites, according to the heatmap and cluster analysis (Figure 2). A slight similarity between the two Latvian sites was mainly associated with low levels of Mg, P, and Cu in both soils. 

**Figure 3:**
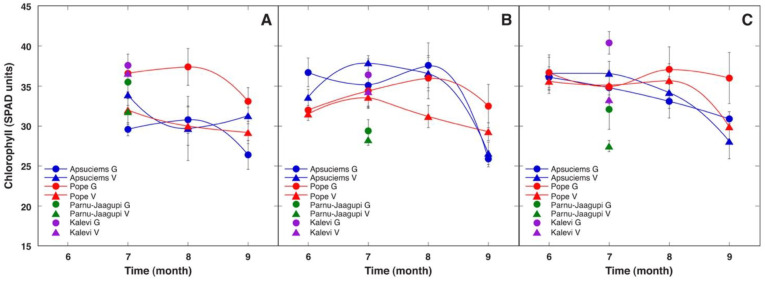
Changes in leaf chlorophyll content in generative (G) and vegetative (V) individuals of *Saussurea esthonica* in field conditions in different study sites in 2009 (**A**), 2010 (**B**), and 2011 (**C**). Data are means ± SE from measurement of 8 flowering and 7 vegetative plants for each time point.

Only relatively minor changes in leaf chlorophyll content were evident during the vegetation season with a tendency to decrease in September (Figure 3). Statistically significant differences in chlorophyll content were evident only in 2009 between generative plants in Apšuciems vs generative plants in Pope, as well as between generative and vegetative plants in Pope (Table S1). Generative plants tended to have a higher leaf chlorophyll concentration in comparison to that in leaves of vegetative plants. When various vegetation seasons were compared with respect to their leaf chlorophyll concentration, no significant differences were seen except for a lower level of chlorophyll in plants in Apšuciems and vegetative plants in Pope in 2009 (Figure 3). In contrast, *S. esthonica* plants in Pärnu-Jaagupi had a lower leaf chlorophyll concentration in 2010 and 2011. In addition, statistically significant differences were observed between the chlorophyll concentration of generative plants in Apšuciems in 2009 and both vegetative and generative plants in Apšuciems in 2010 and 2011.

**Figure 4:**
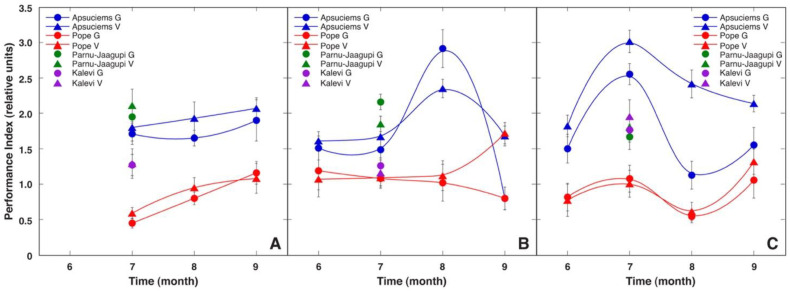
Changes of chlorophyll *a* fluorescence parameter Performance Index in generative (G) and vegetative (V) individuals of *Saussurea esthonica* in field conditions in different study sites in 2009 (**A**), 2010 (**B**), and 2011 (**C**). Data are means ± SE from measurement of 8 flowering and 7 vegetative plants for each time point.

As a maximum quantum efficiency of PSII (F_v_/F_m_) below 0.8 indicates possible photoinhibition of photosynthesis due to unfavorable conditions, it is a useful indicator for recent environmental stress. *S. esthonica* plants in Pope had statistically significant lower F_v_/F_m_ (below 0.8) within all seasons in 2009 as well as in June and July of 2011, when compared to plants in Apšuciems (Figure 4, Table S1). A decrease in the F_v_/F_m_ below 0.8 was noticed in September 2010 for plants in Pope as well as for generative plants in Apšuciems. In August 2011, low F_v_/F_m_ was evident for plants in Pope and generative plants in Apšuciems (Figure 4C). Plants from both populations in Estonia had the same or slightly higher F_v_/F_m_ than that for plants in Apšuciems. Comparing vegetative and generative plants, the parameter F_v_/F_m_ had a tendency to be higher in vegetative plants (Figure 4). A significant difference between vegetative and generative plants was found in July 2009 in Pope (Figure 3A), August 2010 in Pope, September 2010 in Apšuciems (Figure 3B), and in August 2011 in Apšuciems (Figure 4C).

**Figure 5:**
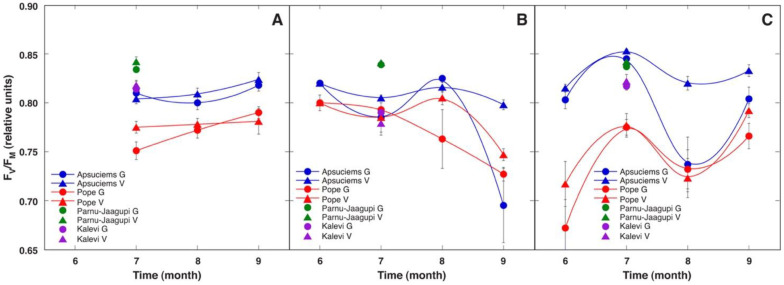
Changes in chlorophyll *a* fluorescence parameter F_v_/F_m_ in generative (G) and vegetative (V) individuals of *Saussurea esthonica* in field conditions in different study sites in 2009 (**A**), 2010 (**B**), and 2011 (**C**). Data are means ± SE from measurement of 8 flowering and 7 vegetative plants for each time point.

**Figure 6:**
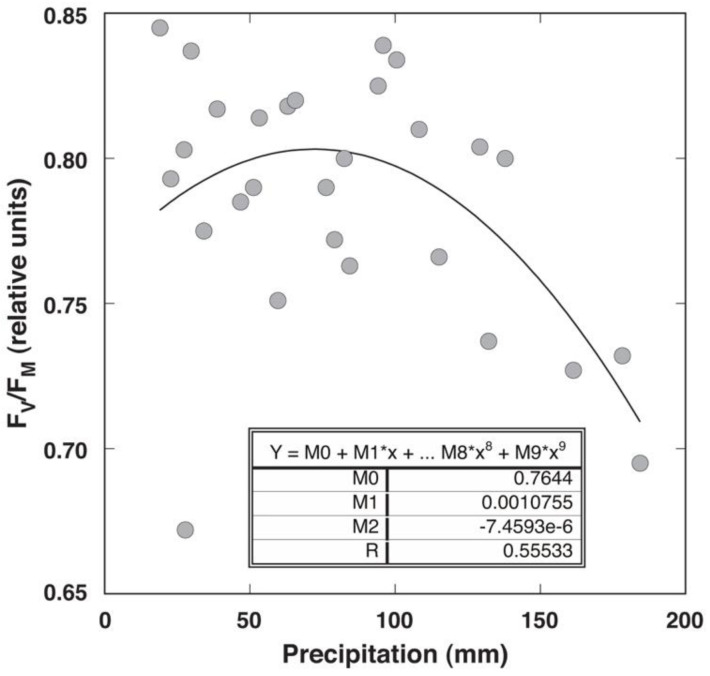
Relationship between chlorophyll *a* fluorescence parameter F_v_/F_m_ in leaves of *Saussurea esthonica* and summary precipitation in the previous month in field conditions.

Changes in the complex fluorescence parameter Performance Index (PI), which characterizes the overall physiological vitality of plants, showed a trend similar to that of F_v_/F_m_, but with less variability at lower values and larger variability at higher values (Figure 5). During the vegetation season of 2009, no drastic fluctuations in PI were observed. However, in August 2010 and June to August 2011, the PI for the plants in Apšuciems increased significantly (Table S1). All measurements of PI in plants from Pope showed statistically significantly lower values than those from Apšuciems plants, except in September 2010. Differences in the PI between generative and vegetative individuals were not statistically significant, except in August 2010 in Apšuciems, September 2010 in Apšuciems and Pope, as well as throughout the vegetation season of 2011 in Apšuciems. Monthly values of the PI correlated with the summary precipitation in the respective month before measurements for a particular site (Figure 6). The correlation corresponded to an exponential regression (*r* = 0.56). There was no significant correlation between the measured physiological parameters and other environmental variables.

Changes in F_v_/F_0_ in *S. esthonica* plant leaves within seasons and between sites were similar to those in the PI, but with a significantly smaller amplitude (data not shown). Changes in the parameters characterizing the proportion of active reaction centers, RC/ABS, were similar to changes in the PI and F_v_/F_0_ (data not shown). The observed changes in RC/ABS were larger in amplitude than F_v_/F_0_, but smaller than changes in the PI. Typically, RC/ABS was higher in plants from Apšuciems than from Pope. In contrast, relatively small differences were observed in the fluorescence parameter measuring dark reaction effects on PS II activity, (1–V_j_)/V_j_ (data not shown).

### 3.2. Effect of Mineral Nutrition

**Figure 7:**
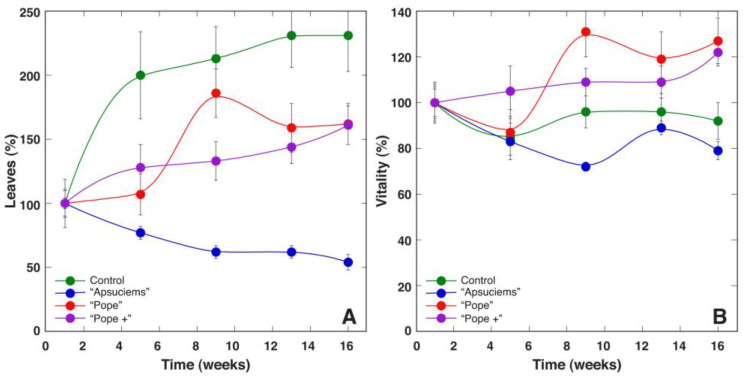
Time course of number of leaves (**A**) and relative vitality (**B**) of *Saussurea esthonica* plants cultivated in substrate with different mineral nutrient composition. Data are means ± SE from 10 plants per treatment.

*S. esthonica* plants grown in control substrate developed relatively more leaves per plant during the experiment, and there were significant differences in this respect from the plants cultivated in “Apšuciems” and “Pope” substrates (Figure 7A, Table S2). Changes in the number of living leaves for plants in “Apšuciems” substrate showed a negative trend during the experiment. Relatively small differences in the visually estimated plant vitality index were found between plants grown in different substrates (Figure 7B). Statistically significant differences in vitality were found only between plants cultivated in control vs “Pope+” substrate, as well as in “Apšuciems” plants vs plants in both “Pope” and “Pope+” substrates (Table S2).

The biomass of the leaves and roots were the lowest from *S. esthonica* plants cultivated in “Apšuciems” substrate and the highest in “Pope+” substrate (Table 3). The difference in leaf biomass between plants in “Pope” and “Pope+” substrate was statistically significant, but the biomass of roots did not differ.

**Figure 8:**
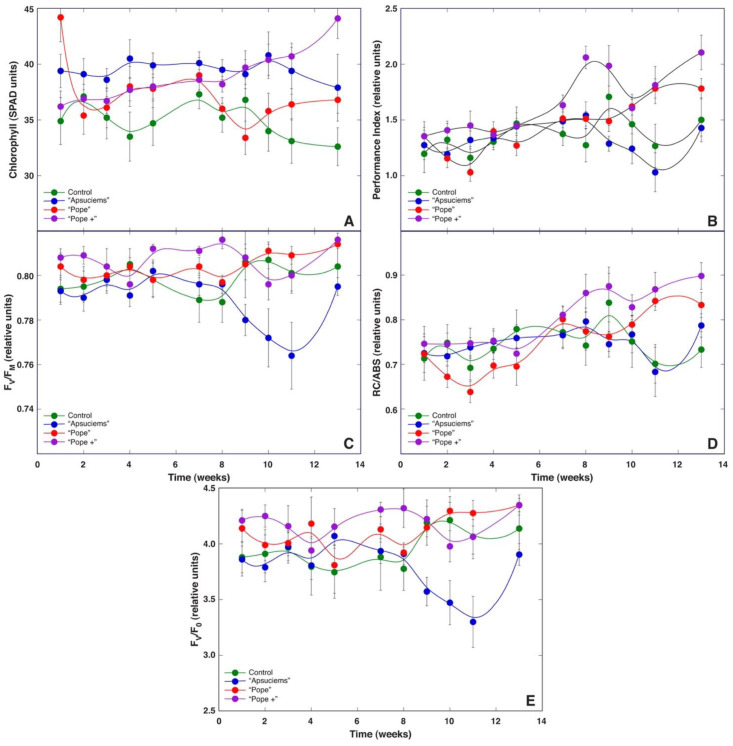
Time course of leaf chlorophyll content (**A**), chlorophyll *a* fluorescence parameters Performance Index (**B**), F_v_/F_m_ (**C**), RC/ABS (**D**), and F_v_/F_0_ (**E**) in *Saussurea esthonica* as affected by different substrate mineral nutrient compositions. Data are means ± SE from measurement of 3–5 leaves per plant from 5 plants per treatment.

With respect to leaf chlorophyll content, relatively small changes during the experiment were found for plants cultivated in “Apšuciems” substrate (Figure 8A). In “Pope” substrate, plants had two periods of a decrease in chlorophyll content—at the very beginning as well as after 7 to 9 weeks—followed by a recovery. In contrast, plants in “Pope+” substrate exhibited a continuous rise in leaf chlorophyll content throughout the experiment, being more intense at the later stage. The leaf chlorophyll content in control plants during the experiment was variable, tending to decrease at 4 weeks as well as from week 10. Statistically significant differences in leaf chlorophyll content were found between plants cultivated in control vs “Apšuciems” substrate, control vs. “Pope”, control vs. “Pope+”, and “Apšuciems” vs “Pope” (Table S3). At the end of the experiment, “Pope+” plants had the statistically significant highest leaf chlorophyll content, followed by identical levels for plants in “Apšuciems” and “Pope” substrates, while control plants had a significantly lower chlorophyll level.

Relatively small changes in chlorophyll *a* fluorescence parameters were observed during the first half of the experiment (Figure 8B–E) except for a slight but statistically significant reduction in PI and RC/ABS up to week 3 for plants in “Pope” substrate. Larger differences in fluorescence parameters were seen in the second half of the study. A summary of statistically significant differences is provided in Table S3. Similar changes in the PI and RC/ABS were observed for *S. esthonica* plants both in “Pope” and “Pope+” substrates, with a near-linear increase in these parameters from week 5 to week 7 (“Pope”) or week 8 (“Pope+”), followed by a further increase in the parameters later on. A decrease in the PI and RC/ABS was evident from week 9 for plants in control and “Apšuciems” substrate, with some recovery at week 13. Less pronounced changes were observed for F_v_/F_m_ and F_v_/F_0_, except for a faster increase in these parameters in “Pope+” plants in the middle part of the experiment, as well as a significant decrease in these parameters in “Apšuciems” plants from week 9 to 11. In general, absolute levels of the PI at the end of the experiment reflected those of leaf number and vitality for plants in the respective substrates.

**Figure 9:**
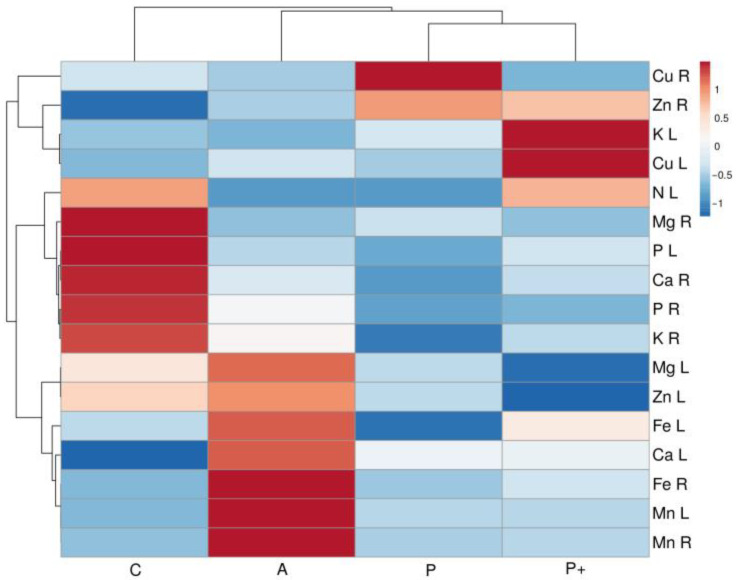
Heatmap and cluster analysis of mineral nutrient content in leaves (L) and roots (R) of *Saussurea esthonica* plants cultivated in substrate with different mineral nutrient compositions. C, control substrate; A, “Apšuciems” substrate; P, “Pope” substrate; P+; “Pope+” substrate.

Differences in leaf and root mineral nutrient concentrations of *S. esthonica* plants in part reflected differences in substrate mineral concentrations. The concentration of N in leaves improved in “Pope+” substrate with the addition of N up to the control level (Table 4). However, the concentration of N was lower in “Apšuciems” treatment despite a higher substrate N concentration. A low substrate P level (50% from control) in all experimental treatments was reflected by a lower leaf P concentration in comparison to control. The leaf K concentration was relatively low in all substrates, but it was higher for plants in “Pope” and “Pope+” treatments despite a lower substrate K concentration when compared to control. Higher amounts of Fe accumulated in “Apšuciems” plants, evidently because of a more than 10-fold higher substrate Fe concentration, but this increase was only 55% in leaves but 148-fold in roots. Multivariate analysis showed that *S. esthonica* plants cultivated in substrates with different mineral nutrient compositions had a rather unique spectrum of mineral elements, with the highest similarity between “Pope” and “Pope+” plants (Figure 9). 

### 3.3. Effect of Soil Moisture

**Figure 10:**
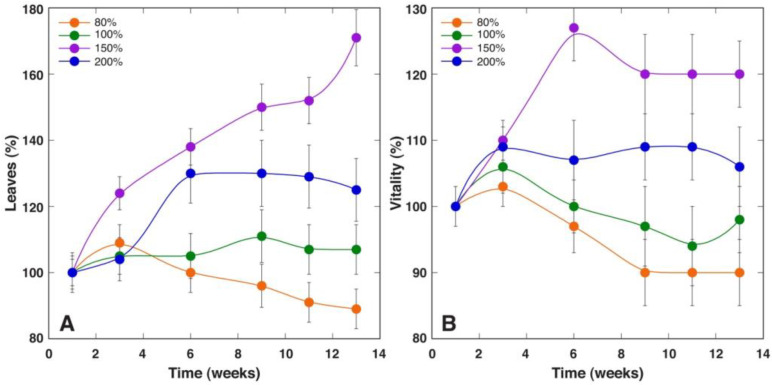
Time course of number of leaves (**A**) and relative vitality (**B**) of *Saussurea esthonica* plants cultivated in substrate with different water availability. Data are means ± SE from 5 plants per treatment.

To evaluate effect of soil moisture on the growth and photosynthesis-associated parameters of *S. esthonica*, plants were cultivated in “Pope+” substrate at four different moisture treatments (dry, normal, wet, and waterlodged). Plant growth during the course of the experiment was most intense in the wet soil (150%) as reflected by changes in the number of leaves per plant (Figure 10A and Figure S3). The number of leaves also increased in waterlodged soil (200%), and remained at a near constant level in normal soil (100%). In contrast, the number of leaves decreased in dry soil (80%). Statistically significant differences in the number of leaves were found between all treatments except wet vs waterlodged treatments (Table S4). Similarly, the vitality of individual plants was the highest at 150% soil moisture, followed by 200%, 100%, and 80% (Figure 10B). However, waterlodged *S. esthonica* plants showed clear symptoms of metabolic disturbance after 4 weeks of cultivation, as indicated by purple spots and regions on leaves between main veins as well as chlorotic lesions at later stages. Statistically significant differences in the vitality index were found between all treatments except dry vs normal treatments (Table S4).

Nevertheless, the biomass of leaves and roots increased with increasing substrate moisture, and all differences between the treatments were statistically significant (Table 5). The biomass of leaves increased by 106% and that of roots by 72% as soil moisture increased from 80% to 100%. For waterlodged plants, leaf biomass increased 263% and root biomass increased by 566%, in comparison to that for plants cultivated at 80%. 

**Figure 11:**
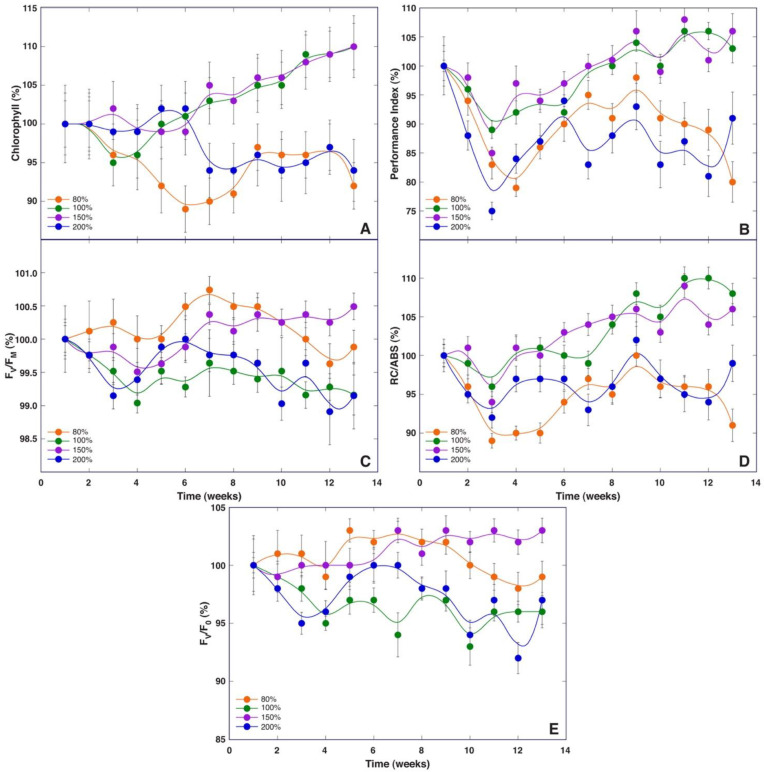
Time course of leaf chlorophyll content (**A**), chlorophyll *a* fluorescence parameters Performance Index (**B**), F_v_/F_m_ (**C**), RC/ABS (**D**), and F_v_/F_0_ (**E**) in *Saussurea esthonica* as affected by different substrate water availability. Data are means ± SE from measurement of 3–5 leaves per plant from 5 plants per treatment.

Leaf chlorophyll concentration did not change significantly within the first six weeks for *S. esthonica* plants growing at 100%, 150%, and 200% moisture treatment (Figure 11A). However, a significant decrease in chlorophyll concentration was noted after 4 weeks for plants grown in dry soil (80%). A near-linear increase in chlorophyll concentration was evident for plants at 100% and 150% soil moisture treatment starting with week 7. At the same time, chlorophyll in leaves of waterlodged plants significantly decreased. At the end of the experiment, plants at 100% and 150% treatments showed the best overall increase in leaf chlorophyll concentration, while plants grown in dry or waterlodged soil performed identically worst. There were no statistically significant differences in leaf chlorophyll content between plants in dry vs waterlodged conditions as well as between optimal and wet conditions (Table S5).

A decrease in both the PI (Figure 11B) and RC/ABS (Figure 11D) at the beginning of the experiment was characteristic for *S. esthonica* plants at all soil moisture levels (up to 3 weeks). After that, these parameters continuously increased for 100% and 150% moisture treatments, while for 80% and 200% treatments, no significant changes were visible after partial recovery for the next three weeks. In contrast, changes in F_v_/F_m_ (Figure 11C) and F_v_/F_0_ (Figure 11E) were less pronounced for all treatments. While certain fluctuations were evident during the experiment, they usually were not statistically significant due to the high individual variability of the parameters. Results of the statistical analysis are provided in Table S5.

A comparison of soil mineral nutrient concentration after cultivation of *S. esthonica* plants at different substrate water availability levels reflected the stimulated uptake and use of mineral elements as a result of growth activation by increased soil moisture (Table 6). This was especially pronounced for N, P, K, S, and Mn.

**Figure 12:**
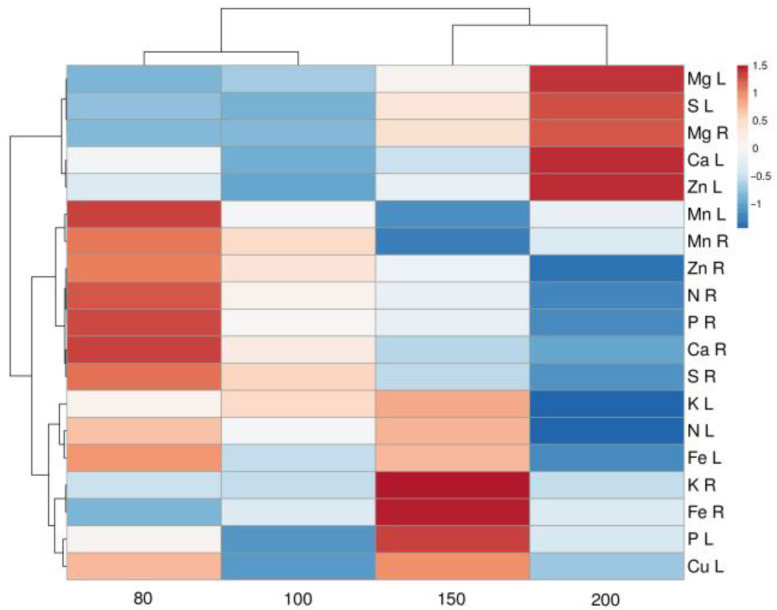
Heatmap and cluster analysis of mineral nutrient content in leaves (L) and roots (R) of *Saussurea esthonica* plants cultivated in substrate with different water availability.

The soil moisture regime significantly affected the concentration of several macro- and micronutrients in the leaves and roots of *S. esthonica* plants (Table 7). Thus, the concentration of N, K, Ca, S, and Zn in roots gradually decreased with the increase in substrate moisture. In leaves, an increase in concentration with increasing substrate moisture was evident for Mg and S. Other elements did not show clear causal changes in concentration. Multivariate analysis revealed that mineral nutrient composition was relatively similar between plants from dry and optimal treatments, and with less similarity between those from wet and waterlodged conditions (Figure 12).

## 4. Discussion

### 4.1. Evaluation of Physiological Status

In the case of studies with rare and endangered plants, especially with respect to legally protected species, special care is necessary to avoid any disturbances and harm to the native populations. In natural conditions, chlorophyll *a* fluorescence-derived parameters have been shown to be reliable indicators of the physiological state of rare plant populations [6,17,18,19,20]. However, to understand a possible degree of generalizability of such results, it is necessary to point out that (i) the results cannot be easily compared between different genotypes and sites with different sets of environmental conditions, (ii) the growth and physiological indices do not always correlate tightly, and (iii) changes in various chlorophyll *a* fluorescence parameters may have different physiological meaning. These important restrictions need to be discussed in some detail in the context of the present study.

The first restriction has been previously thoroughly discussed in a specialized literature [30]. Most importantly, it must be considered that fluorescence data can be used only for the comparison of the impact of certain environmental factor(s) on the photochemistry of photosynthesis, but any absolute values have no physiological meaning. Therefore, genetically different plant populations can be evaluated only with respect to the impact of some environmental factors, rather than a direct comparison of their fluorescence parameter values. The only partial exception is for F_v_/F_m_, representing a maximum quantum yield of photochemistry of photosystem II, which is around 0.8 for plants in conditions near a physiological optimum, and diminishes in unfavorable conditions most likely related to the photoinhibition of photosynthesis through direct damage to D1 proteins [31]. The chlorophyll fluorescence ratio F_v_/F_m_ in *S. esthonica* plants at the Pope site was below 0.8, indicating photoinhibition of photosynthesis. In turn, in the Apšuciems site, this ratio was slightly above 0.8 (except for in August 2011 in the generative plants). This was probably due to increased precipitation in Pope, because as rainfall increases, photosystem II activity-related indicators decrease (Figure 6). The sharp decrease in F_v_/F_m_ in Pope in August 2011 could be due to increased precipitation. A similar negative relationship between diminished F_v_/F_m_ and increased summary precipitation in the previous months has been found for a the coastal-specific endangered species *Eryngium maritimum* [8]. However, *E. maritimum* is a xerophytic drought-tolerant species well adapted to the hot Mediterranean conditions, and these plants have a relatively low vitality in Northern Europe, while *S. esthonica* seems to be well adapted to high substrate moisture—even requiring it for optimal growth.

While all chlorophyll fluorescence-derived parameters obtained through fast induction kinetics are interlinked, each of them shows effects at specific points in the electron transport chain [30]. F_v_/F_0_ is mostly affected by photochemical reactions at the donor side of photosystem II, including water-splitting activity [30], and changes in this parameter have been shown to be an important determinant of biomass accumulation of *Dracocephalum moldavica* plants at various fertilization regimes [32]. However, F_v_/F_0_ did not show a direct relationship with growth at different mineral regimes in *S. esthonica* (Figure 8E) or biomass accumulation in plants at different soil moisture regimes (Figure 11). Instead, both the Performance Index and RC/ABS showed better resolution for biomass accumulation, separating treatments with good vs poor growth (Figure 8B,D and Figure 11B,D). Consequently, fluorescence emission on the absorption basis together with energy fluxes related to a reduction in end electron acceptors of photosystem I [30] are important indicators of biomass accumulation in *S. esthonica*.

It is suggested that changes in the chlorophyll concentration in plants reflects the impact of relatively long-term environmental changes [33]. However, even a relatively drastic decrease in leaf chlorophyll content usually has only minor negative effects on the photochemical activity of photosystem II [34,35]. In the present study, at the end of the vegetation season, chlorophyll concentration tended to decrease, which can be explained by the aging of leaves [35] The chlorophyll concentration in flowering individuals was larger than that in the leaves of vegetative individuals in the Pope and Estonian populations, however, in the Apšuciems population, the opposite relationship was observed. A study of chlorophyll in leaves of various rose species and varieties found that in most cases, the amount of chlorophyll decreases during flowering and increases during the fruit-ripening stage [36]. A study of corn hybrids found that after flowering, chlorophyll concentration and F_v_/F_m_ was reduced in most cases as a result of aging [37]. In the present study, measurements in July correspond to the flowering phase, while the August measurements correspond to the seed-maturation phase. In addition, it was observed that, somehow, the Pope plants flowered longer, and some plants were still flowering in August. The variation in the flowering dynamic was also observed between different seasons. In general, chlorophyll concentration increased slightly between flowering and fruit ripening, however, in 2011, this increase was less pronounced in the Apšuciems population. The leaf chlorophyll concentration of vegetative individuals began to decrease as early as July (except in 2011 in the Apšuciems population) (Figure 1). In senescing leaves, chlorophyll concentration decreases, since metabolites diffuse to reaction centers, and the chlorophyll synthesis rate decreases [35]. Consequently, the present study shows that the amount of chlorophyll depends on both environmental and ontogenesis factors.

It is evident that photosynthesis-related parameters, chlorophyll concentration, and chlorophyll *a* fluorescence-derived parameters do not always perfectly correlate with the growth performance of plants. Therefore, special care needs to be taken for the complex evaluation of all available indices in order to predict the physiological performance of model plants.

### 4.2. Effect of Mineral Nutrition

The presence of several *Saussurea* species in calcareous soils indicates a good adaptability to high bicarbonate conditions. These soils are identified by the presence of a high amount of CaCO_3_ and a relatively high pH reaction, and HCO_3_^–^ concentrations in calcareous soils increases with the rise in soil moisture [38]. The growth of carbonate-susceptible species in calcareous soils is significantly reduced together with negative effects on mineral nutrition and photosynthesis at the level of enhancing photoinhibition [39]. With respect to mineral nutrition, Fe deficiency is a common symptom in calcareous soils, resulting in leaf chlorosis [40]. However, calcicole species can control mineral nutrient uptake through local soil acidification and the release of chelating compounds [38]. Two subspecies of *S. alpina* in the Western Alps were found to be natively growing in soils with different pH values: 6.00–7.31 for *S. alpina* subsp. *alpina* and 7.93–8.65 for *S. alpina* subsp. *depressa* [41]. In the present study, the soil pH range found for *S. esthonica* (pH 6.2–7.3) was closer to that of *S. alpina* subsp. *alpina* (Table 2). In the soil containing the highest concentration of Ca (in the Apšuciems site), the highest concentration of Fe was also present (Table 2), but *S. esthonica* plants did not show any signs of photoinhibition of photosynthesis, as F_v_/F_m_ values tended to be higher than in the site with a lower soil Ca level (Figure 5).

It was initially hypothesized that *S. esthonica* plants can adapt to a relatively wide range of soil mineral nutrient concentrations. Indeed, the concentration of plant-available mineral elements in the natural soils of *S. esthonica* was highly variable between the sites, with especially high variability for Ca, Mg, S, Fe, and Mn (Table 2). Several times higher concentrations of Fe, S, and Mn in the native soil of the Apšuciems site could be associated with continuously expressed higher performance of Apšuciems plants with respect to the photochemistry of photosynthesis (Figure 4 and Figure 5). The functional basis of this effect is related to the fact that all these elements are important for light-dependent reactions of photosynthesis: Fe is a cofactor in photosynthesis complexes and participates in many components of the photosynthetic electron transport chain, Mn is a functional component of a water-splitting complex of photosystem II, and Fe–S clusters are integral structural components of photosystem I reaction centers, cytochrome b6f, and ferredoxin [42].

Therefore, we decided to establish four substrates with different mineral nutrient concentrations for a study in controlled conditions, based on the composition that is optimal for a majority of crop plants (control), soil in Apšuciems site (“Apšuciems”), soil in Pope site with lower N, Mg, S, Fe, Mn, and B than in “Apšuciems” soil (“Pope”), and soil based on the previous soil but with increased N and S concentrations (“Pope+”). Surprisingly, plants in “Apšuciems” soil had very low growth (Table 5) and suppressed development (Figure 7A), and chlorophyll fluorescence-related parameters significantly decreased in these plants at the last phase of cultivation (Figure 8). However, the chlorophyll content in the leaves of plants in “Apšuciems” soil was stable and high throughout the cultivation period (Figure 8A). Consequently, some other factors in the native site of *S. esthonica* at Apšuciems were responsible for the increased photochemical performance of photosynthesis, both physicochemical and biological. It is possible that this discrepancy was related to differences in photosynthetically active radiation intensity between natural and controlled conditions, which did not allow for positive effects in low-light conditions, as many plant responses to single factors differ to those in presence of additional factors [43]. However, an increase in N and S concentrations in “Pope+” substrate in comparison to those in “Pope” substrate significantly improved shoot growth (Table 3) and increased leaf chlorophyll content, but not chlorophyll fluorescence indices of *S. esthonica* plants. 

### 4.3. Effect of Soil Moisture

In a vegetation study performed in Estonia, *S. esthonica* plants were located in the part of the calcareous fen with the shallow depth to water level, characterized by high electrical conductivity, but multivariate analysis did not show any significant association between the presence of the individuals and any environmental variables [44]. However, it was suggested that *S. esthonica* can benefit from increasing shade due to the presence of shrubs and sparse trees [44]. *Cladium mariscus*, another typical but extremely rare species of calcareous fens, did not show any immediate effect of habitat water level changes on the growth of generative shoots [45]. Seasonal measurements of F_v_/F_m_ indicated good adaptability of the species to high substrate water level, while being sensitive to a decrease in the water level below 5 cm [45].

Soil moisture has a substantial influence on soil chemistry, leading to differences in mineral nutrient availability and uptake in plants [38,46]. However, the response is usually genotype-specific, as significant differences were found in mineral nutrient responses to increasing soil moisture in different species including *Veronica spicata* and *Phleum phleoides* [38]. Thus, shoot Ca, K, P, and Mg concentration increased only in *Veronica spicata* plants with increasing soil moisture levels, with no significant changes in *Phleum phleoides*. However, only the concentrations of Mg and S gradually increased with increasing soil moisture in leaves of *S. esthonica* plants (Table 7).

*S. esthonica* is closely related, both morphologically and genetically, to other species of the genus including *S. alpina* and *Saussurea discolor* [12,13]. However, the results obtained in the present study are difficult to compare with those obtained with taxonomically related *Saussurea* species due to the rather unique ecological niche of *S. esthonica*. *Saussurea salsa*, a species from salt marshes, showed morphological changes in leaves as a part of the adaptation strategy of plants from low to deep flooding, involving an increase in specific leaf area, together with a plasticity of photochemical reactions of photosynthesis [47]. This was similar to the results of the present study, showing that waterlodged conditions were well tolerated up to 6 weeks, as indicated by stable trends both in the amount of leaf chlorophyll and indices of photochemistry of photosynthesis, followed by a decrease in the later stages of cultivation (Figure 11). The optimal growth of *S. esthonica* plants was evident in waterlodged conditions, decreasing with a decrease in soil moisture (Table 5, Figure 10), but physiological performance, as indicated by chlorophyll levels and the chlorophyll fluorescence parameters Performance Index and RC/ABS, was better for plants at moderate- (100%) and high- (150%) moisture conditions (Figure 11). However, the maximum quantum efficiency of photosystem II (F_v_/F_m_), an indicator of photoinhibition of photosynthesis, showed that only at 150% moisture plants showed no signs of metabolic disturbance. Therefore, *S. esthonica* plants can be characterized as a moisture-demanding species, with extremely high tolerance to soil waterlodged at the level of growth and photosynthesis. Other relatively waterlodged-tolerant species, such as *Trifolium fragiferum*, usually show chlorosis and other signs of metabolic disturbance even after several weeks in waterlodged conditions [48]. In *S. esthonica*, certain visual signs of metabolic disturbances due to waterlodging appeared in the form of purple spots after 4 weeks and in the form of chlorotic lesions only after 12 weeks.

### 4.4. Future Perspectives

Calcareous fens have been recognized as local hotspots of biological diversity and refugia for rare and endangered plant species [49]. A set of specific environmental conditions is responsible for the development of unique vegetation in calcareous fen habitats, but also determines its high vulnerability. In particular, alkaline fens are highly dependent on the inflow of calcareous groundwater resulting in a stable near-surface water level [50]. Seasonal fluctuations in the water level occur mainly due to the differences in precipitation. The composition of plant species in calcareous fens depends on the amplitude of seasonal water level fluctuations, and changes less than 20–25 cm are suggested to be optimal for calcicole vascular plant species [44]. Results of the present study suggest that *S. esthonica* is strongly dependent on high substrate moisture. Therefore, the preservation of the natural water regime in the natural habitats of *S. esthonica* is critical to the conservation of the species.

Genetic differentiation of *S. esthonica* plants between the two Latvian sites, Apšuciems and Pope, was only 3–5%, pointing to their common provenance [51]. However, there was a 10–13% differentiation between the two Latvian populations and those from Estonia (Pärnu-Jaagupi and Kalevi), but genetic polymorphism was lower in the Estonian populations. Therefore, in future studies, it will be necessary to compare the response of *S. esthonica* plants from Latvian and Estonian populations to changes in environmental factors under controlled conditions, in order to find out whether genetic differences are related to differences in the ability of plants to adapt to a changing environment.

## 5. Conclusions

In the studies of rare and endangered plant species, it is necessary to avoid any disturbance and harm to the native populations. In native conditions, the physiological status of *S. esthonica* is affected both by the soil chemical composition and precipitation, but other environmental factors, such as temperature and light regime, could be important. In controlled conditions, substrate drying had more negative effects on the growth of *S. esthonica* plants in comparison to that of waterlodging, and this can be directly linked to prevalent hydrological conditions of the alkaline fen habitat which is native to the species. As the growth and physiological performance of *S. esthonica* strongly depends on high substrate moisture, the preservation of the natural water regime in the natural habitats is critical to the conservation of the species.

## Figures and Tables

**Table 1:** Substrate mineral nutrient concentrations used (mg L^–1^) in the mineral nutrient experiment in controlled conditions.

Element	Control	“Apšuciems”	“Pope”	“Pope+”
N	120	126	61	120
P	155	74	74	74
K	390	155	155	155
Ca	14,800	13,167	13,167	13,167
Mg	5450	2306	300	300
S	120	250	22	120
Fe	265	3983	240	240
Mn	45	883	38	38
Zn	1.5	7	7	7
Cu	2.20	1.65	1.65	1.65
Mo	0.04	0.05	0.05	0.05
B	0.10	2.03	1.33	1.33
Na	30	30	30	30

**Table 2:** Characterization of soil at *Saussurea esthonica* sites in Latvia (Apšuciems, Pope) and Estonia (Pärnu-Jaagupi, Kalevi).

Parameter	Apšuciems	Pope	Pärnu-Jaagupi	Kalevi
N	126 ± 12 ^a^	61 ± 10 ^b^	124 ± 10 ^a^	73 ± 2 ^b^
P	40 ± 2 ^c^	54 ± 16 ^bc^	225 ± 29 ^a^	68 ± 5 ^b^
K	76 ± 17 ^a^	87 ± 22 ^a^	49 ± 4 ^b^	58 ± 10 ^b^
Ca	54,700 ± 6307 ^a^	13,167 ± 3415 ^b^	7267 ± 996 ^d^	9583 ± 910 ^c^
Mg	306 ± 10 ^c^	248 ± 45 ^d^	1805 ± 671 ^a^	747 ± 77 ^b^
S	600 ± 25 ^a^	45 ± 8 ^b^	19 ± 2 ^c^	20 ± 2 ^c^
Fe	5350 ± 400 ^a^	240 ± 33 ^d^	1958 ± 80 ^b^	1581 ± 80 ^c^
Mn	883 ± 233 ^a^	38 ± 6 ^c^	172 ± 22 ^b^	60 ± 17 ^c^
Zn	7.2 ± 0.6 ^a^	7.2 ± 1.5 ^a^	6.3 ± 1.1 ^a^	6.0 ± 0.9 ^a^
Cu	1.4 ± 0.1 ^c^	1.6 ± 0.4 ^bc^	4.8 ± 0.7 ^a^	1.9 ± 0.2 ^b^
Mo	0.06 ± 0.01 ^a^	0.05 ± 0.01 ^ab^	0.04 ± 0.00 ^b^	0.04 ± 0.00 ^b^
B	14.5 ± 1.8 ^a^	2.6 ± 0.6 ^b^	1.3 ± 0.4 ^c^	2.9 ± 0.5 ^b^
Na	46 ± 5 ^a^	23 ± 4 ^b^	n.d.	n.d.
pH_KCl_	7.3 ± 0.0 ^a^	6.6 ± 0.1 ^b^	6.2 ± 0.2 ^b^	6.4 ± 0.1 ^b^
Electrical Conductivity (mS m^–1^)	2.9 ± 0.2 ^a^	0.7 ± 0.1 ^b^	0.7 ± 0.1 ^b^	0.6 ± 0.1 ^b^

Plant-available concentrations are indicated, measured in 1 M HCl extract. Concentrations of mineral nutrients are in mg L^–1^. Electrical conductivity was measured at a 1:5 extraction ratio. Data are means from the analysis of three independent samples ± SD. Values followed by different letters for a particular parameter differ statistically significantly (*p* < 0.05).

**Table 3:** Biomass of leaves and roots of *Saussurea esthonica* plants cultivated in different substrates.

Substrate Type	Leaf Fresh Mass (g plant^–1^)	Root Fresh Mass (g plant^–1^)
Control	1.17 ± 0.10 ^c^	2.45 ± 0.18 ^b^
“Apšuciems”	0.83 ± 0.07 ^c^	1.52 ± 0.16 ^c^
“Pope”	1.78 ± 0.22 ^b^	3.78 ± 0.28 ^a^
“Pope+”	3.33 ± 0.17 ^a^	3.96 ± 0.31 ^a^

Data are means ± SE from 10 plants per treatment. Values followed by different letters for the particular parameters differ statistically significantly (*p* < 0.05).

**Table 4:** Concentration of mineral nutrients in leaves and roots of *Saussurea esthonica* cultivated in different substrates.

Element	Leaves				Roots			
	C	A	P	P+	C	A	P	P+
	Macronutrients (%)							
N	1.70 ^a^	1.25 ^b^	1.25 ^b^	1.67 ^a^	1.30 ^a^	n.d.	n.d.	n.d.
P	0.49 ^a^	0.19 ^c^	0.14 ^c^	0.21 ^c^	0.52 ^a^	0.34 ^b^	0.21 ^c^	0.23 ^c^
K	3.90 ^b^	3.80 ^b^	4.13 ^b^	5.45 ^a^	1.80 ^c^	1.65 ^cd^	1.48 ^d^	1.57 ^cd^
Ca	1.76 ^b^	2.86 ^a^	2.32 ^ab^	2.30 ^ab^	0.87 ^c^	0.64 ^d^	0.55 ^d^	0.62 ^d^
Mg	0.48 ^a^	0.50 ^a^	0.46 ^a^	0.44 ^a^	0.29 ^b^	0.20 ^b^	0.21 ^b^	0.20 ^b^
	Micronutrients (mg kg^–1^)							
Fe	150 ^f^	233 ^d^	113 ^g^	188 ^e^	225 ^d^	1750 ^a^	300 ^c^	467 ^b^
Mn	32 ^f^	2400 ^b^	288 ^cd^	278 ^de^	25 ^f^	3700 ^a^	225 ^e^	317 ^b^
Zn	32 ^cd^	34 ^cd^	27 ^d^	23 ^d^	38 ^c^	55 ^b^	91 ^a^	85 ^a^
Cu	6.5 ^c^	6.7 ^c^	6.3 ^cd^	10.5 ^a^	10.5 ^a^	7.0 ^c^	4.3 ^d^	5.0 ^d^

n.d., not determined. C, control; A, “Apšuciems”; P, “Pope”; P+, “Pope+”. Data are means from analysis of three independent samples. Values followed by different letters for the particular nutrients differ statistically significantly (*p* < 0.05).

**Table 5:** Biomass of leaves and roots of *Saussurea esthonica* plants cultivated in substrate with different moisture.

Moisture Level (%)	Leaf Fresh Mass (g plant^–1^)	Root Fresh Mass (g plant^–1^)
80	1.48 ± 0.12 ^d^	3.53 ± 0.41 ^d^
100	3.05 ± 0.11 ^c^	6.08 ± 0.53 ^c^
150	4.56 ± 0.20 ^b^	10.78 ± 0.58 ^b^
200	5.37 ± 0.27 ^a^	23.50 ± 0.54 ^a^

Data are means ± SE from 5 plants per treatment. Values followed by different letters for the particular parameters differ statistically significantly (*p* < 0.05).

**Table 6:** Concentration of mineral nutrients (mg L^–1^) in substrate after cultivation of *Saussurea esthonica* at different water supply levels in controlled conditions.

Parameter	80%	100%	150%	200%
N	113 ^a^	75 ^b^	67 ^b^	27 ^c^
P	87 ^a^	65 ^b^	52 ^c^	53 ^c^
K	165 ^a^	22 ^b^	16 ^bc^	11 ^c^
Ca	6750 ^a^	6600 ^a^	6900 ^a^	6100 ^b^
Mg	400 ^b^	375 ^b^	535 ^a^	270 ^c^
S	538 ^a^	457 ^b^	425 ^c^	105 ^d^
Fe	665 ^a^	620 ^b^	535 ^c^	575 ^bc^
Mn	90 ^a^	70 ^b^	65 ^b^	33 ^c^
Zn	15 ^a^	16 ^a^	15 ^a^	9 ^b^
Cu	0.9 ^c^	1.1 ^c^	2.4 ^b^	4.8 ^a^
Mo	0.11 ^a^	0.11 ^a^	0.06 ^b^	0.09 ^ab^
B	2.4 ^ab^	2.7 ^a^	2.4 ^ab^	1.8 ^b^

Plant-available concentrations are indicated, measured in 1 M HCl extract. Data are means from analysis of three independent samples. Values followed by different letters for the particular nutrients differ statistically significantly (*p* < 0.05).

**Table 7:** Concentration of mineral nutrients in leaves and roots of *Saussurea esthonica* cultivated in conditions of different water supply.

Element	Leaves				Roots			
	80%	100%	150%	200%	80%	100%	150%	200%
	Macronutrients (%)							
N	1.60 ^a^	1.43 ^b^	1.70 ^a^	0.93 ^c^	1.90 ^a^	1.40 ^b^	1.27 ^bc^	0.80 ^c^
P	0.14 ^bc^	0.09 ^c^	0.19 ^bc^	0.12 ^c^	0.31 ^a^	0.25 ^ab^	0.24 ^ab^	0.19 ^bc^
K	6.10 ^a^	6.55 ^a^	6.95 ^a^	4.22 ^b^	2.40 ^a^	1.87 ^b^	1.60 ^c^	1.02 ^d^
Ca	2.45 ^b^	2.24 ^b^	2.34 ^b^	2.80 ^a^	0.77 ^c^	0.66 ^cd^	0.58 ^d^	0.54 ^d^
Mg	0.30 ^c^	0.32 ^ab^	0.41 ^b^	0.56 ^a^	0.15 ^d^	0.15 ^d^	0.25 ^cd^	0.32 ^c^
	Micronutrients (mg kg^–1^)							
Fe	150 ^c^	80 ^d^	140 ^c^	50 ^d^	300 ^b^	310 ^b^	345 ^a^	310 ^b^
Mn	400 ^d^	290 ^c^	195 ^b^	276 ^c^	520 ^e^	430 ^d^	160 ^b^	304 ^c^
Zn	27 ^e^	23 ^e^	28 ^e^	38 ^d^	87 ^a^	80 ^ab^	75 ^bc^	62 ^c^
Cu	11 ^a^	3.5 ^c^	12 ^a^	5 ^b^	n.d.	n.d.	n.d.	n.d.

n.d., not determined. C, control; A, “Apšuciems”; P, “Pope”; P+, “Pope+”. Data are means from analysis of three independent samples. Values followed by different letters for the particular nutrients differ statistically significantly (*p* < 0.05).

## Data Availability

All data reported here are available from the authors upon request.

## Supplementary Materials

The following supporting information can be downloaded at: https://www.mdpi.com/article/10.3390/plants12040888/s1, Figure S1: *Saussurea esthonica* sites in Latvia and Estonia, used in the present study; Figure S2: Typical *Saussurea esthonica* individuals in mineral nutrient experiments in controlled conditions; Figure S3: Typical *Saussurea esthonica* individuals in water availability experiments in controlled conditions; Table S1: Results of ANOVA analysis of photosynthesis-related parameters of *Saussurea esthonica* between sites, and vegetative (V) and generative (G) individuals; Table S2: Results of ANOVA analysis of the number of leaves and plant vitality index of *Saussurea esthonica* plants cultivated in substrate with different mineral nutrient compositions; Table S3: Results of ANOVA analysis of photosynthesis-related parameters of *Saussurea esthonica* plants cultivated in substrate with different mineral nutrient compositions; Table S4: Results of ANOVA analysis of the number of leaves and plant vitality index of *Saussurea esthonica* plants cultivated in substrate with different water availability; Table S5: Results of ANOVA analysis of photosynthesis-related parameters of *Saussurea esthonica* plants cultivated in substrate with different water availability.
