# Supplementary material for: Ecophysiology of Endangered Plant Species Saussurea esthonica: Effect of Mineral Nutrient Availability and Soil Moisture
Source: Plants (Basel). 2023 Feb 16;12(4):888. doi: 10.3390/plants12040888 (PMC9965748; doi:10.3390/plants12040888)
Supplement: Supplementary file 1 [file plants-12-00888-s001.zip › Saussurea Supplement 1.pdf]

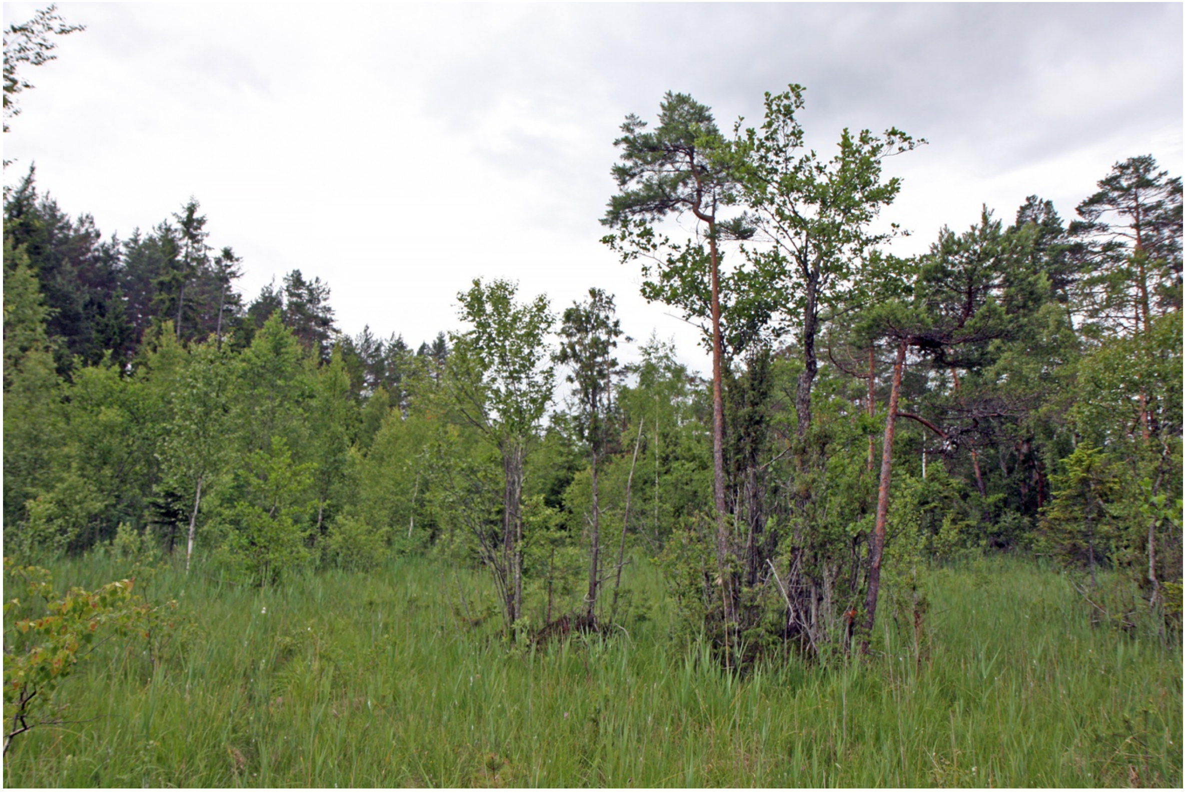

Figure S1. *Saussurea esthonica* sites in Latvia and Estonia, used in the present study.

A, site in Apšuciems, Latvia.

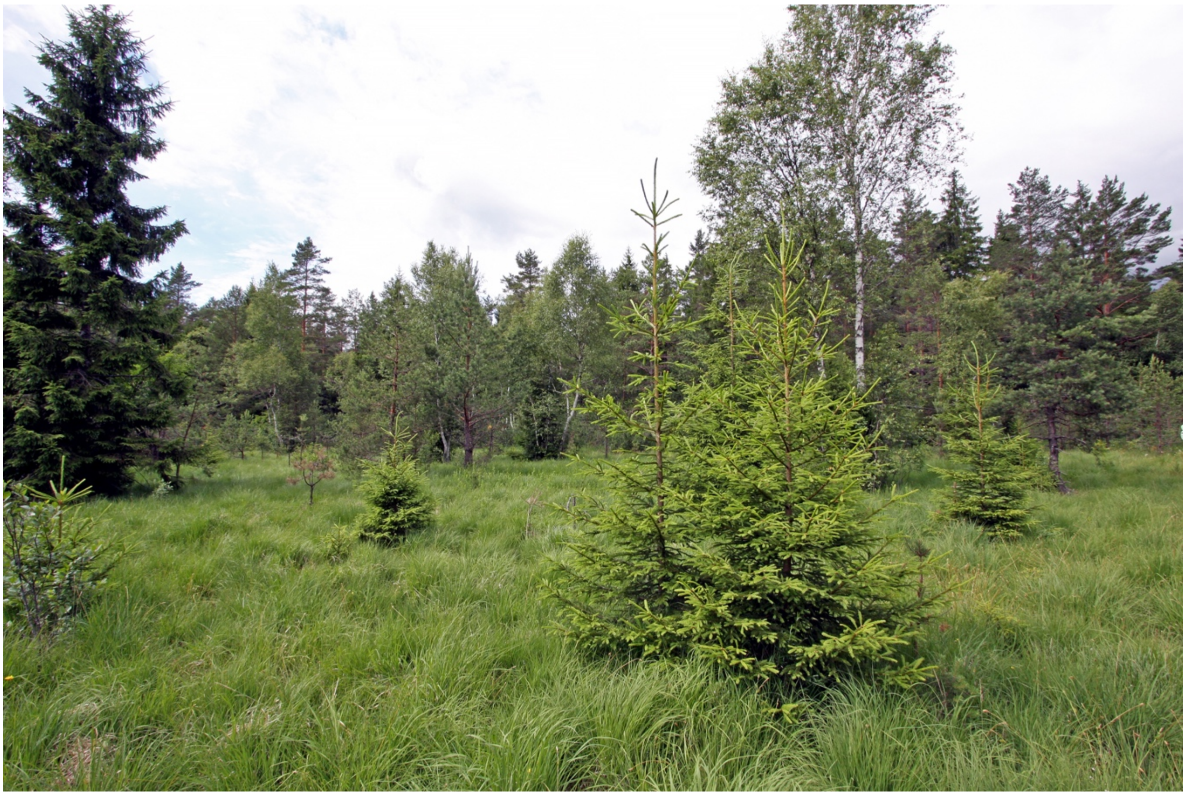

B, site in Pope, Latvia.

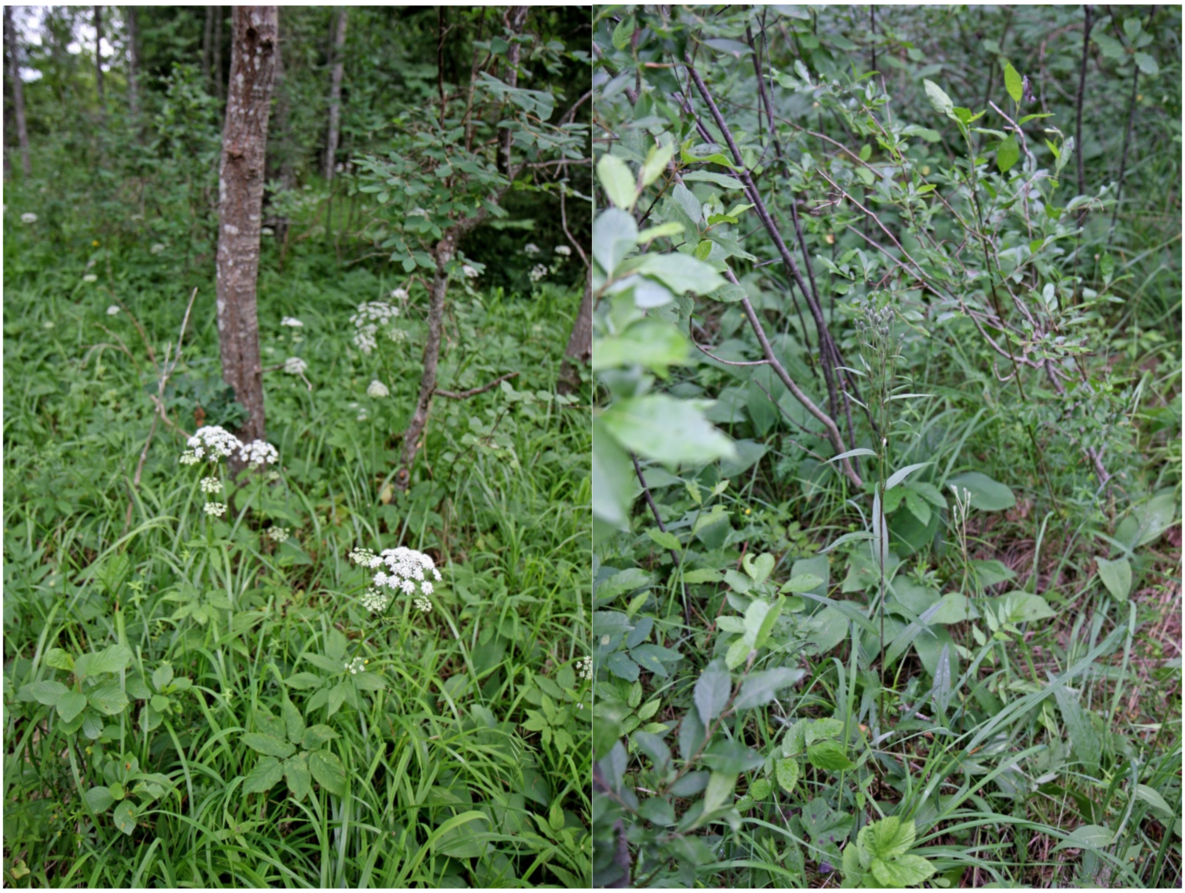

C, site in Pärnu-Jaagupi, Estonia.

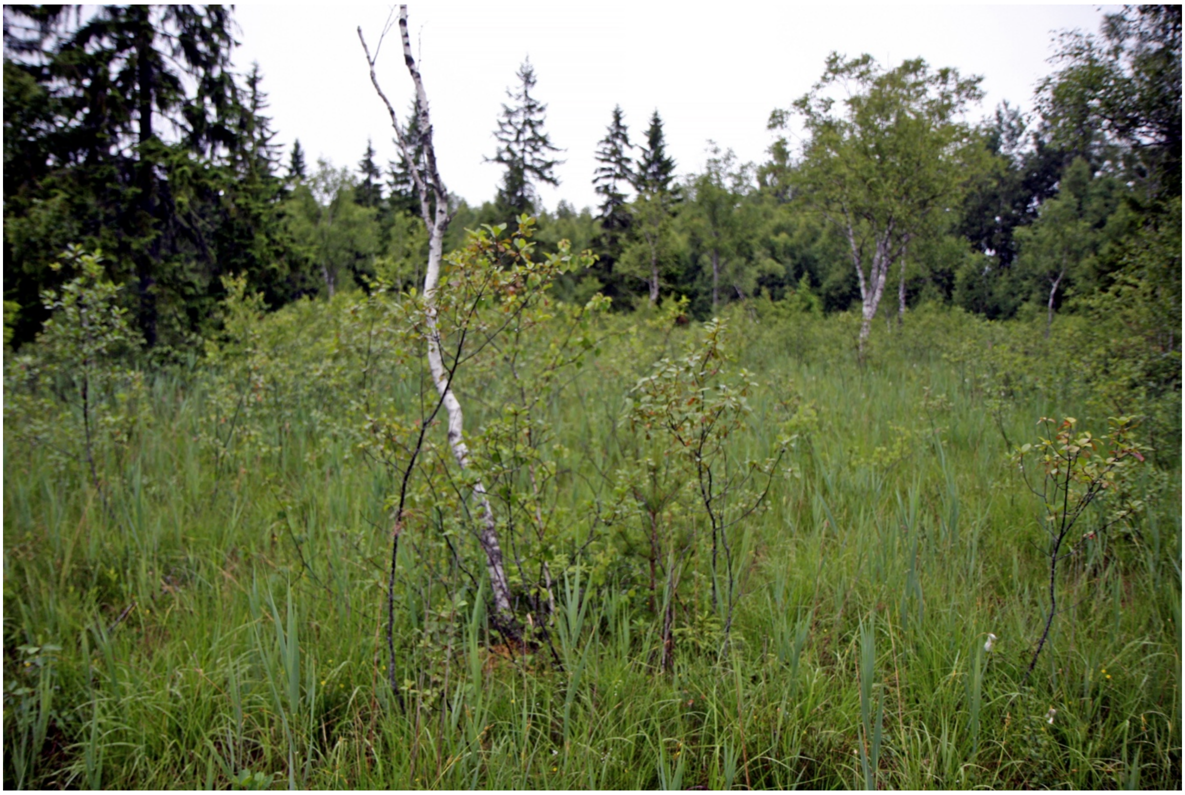

D, site in Kalevi, Estonia.

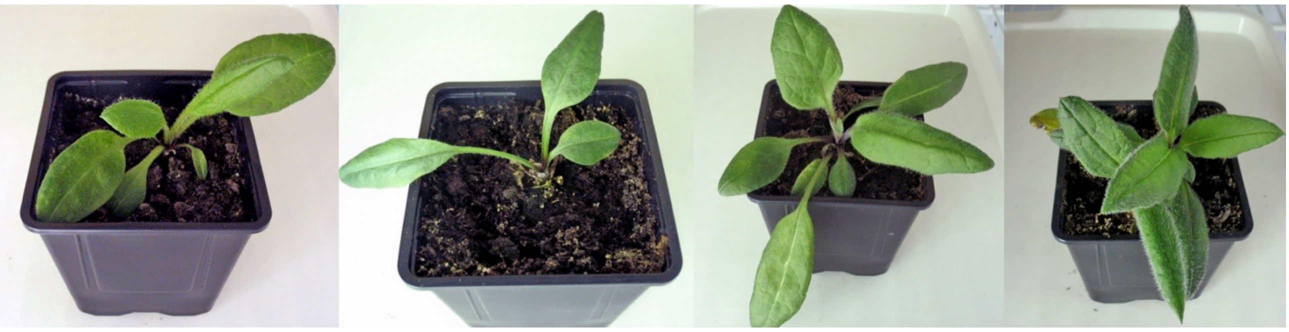

Figure S2. Typical *Saussurea esthonica* individuals in mineral nutrient experiment in controlled conditions. From left to right: control, "Apšuciems", "Pope", "Pope+".

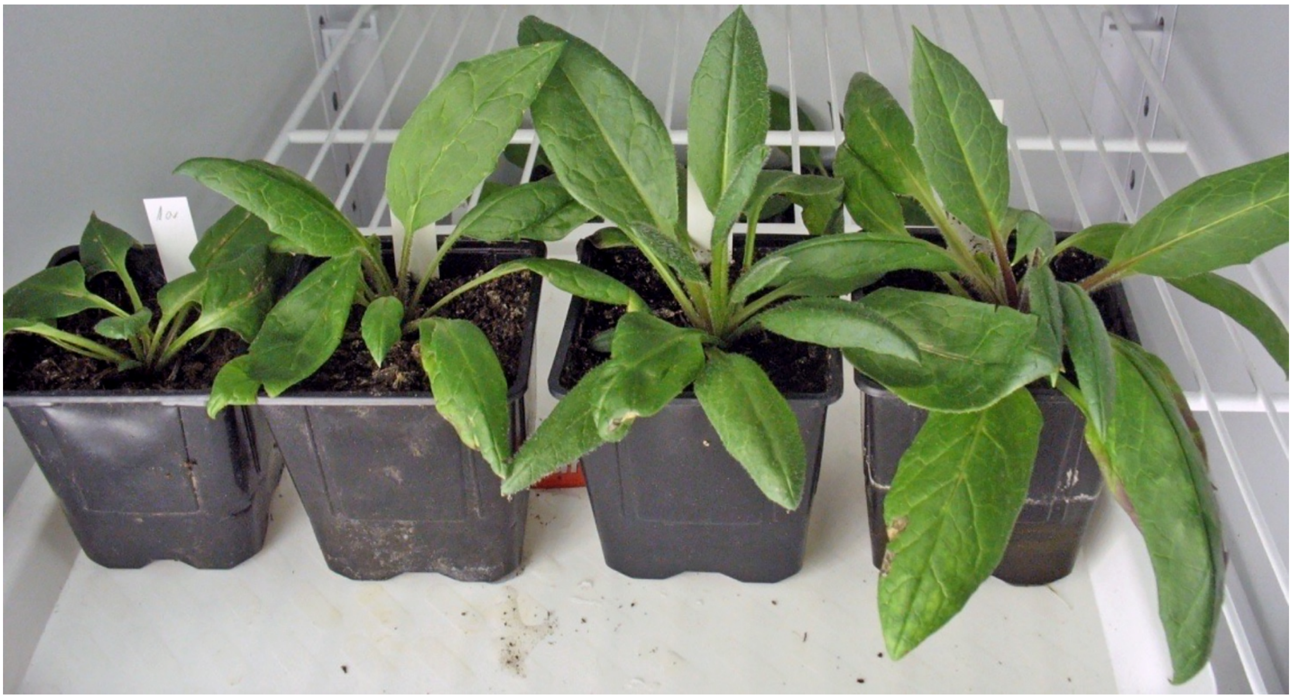

Figure S3. Typical *Saussurea esthonica* individuals in water availability experiment in controlled conditions. From left to right: 80, 100, 150, 200% substrate water content.
