# Supplementary material for: Ecophysiology of Endangered Plant Species Saussurea esthonica: Effect of Mineral Nutrient Availability and Soil Moisture
Source: Plants (Basel). 2023 Feb 16;12(4):888. doi: 10.3390/plants12040888 (PMC9965748; doi:10.3390/plants12040888)
Supplement: Supplementary file 1 [file plants-12-00888-s001.zip › Saussurea Supplement 2.pdf]

Table S1. Results of ANOVA analysis of photosynthesis-related parameters of *Saussurea esthonica* between sites, and vegetative (V) and generative (G) individuals. Statistically significant ($p < 0.05$) interactions are indicated in bold. d.f., degrees of freedom.

Site Interactions	Chlorophyll Content		F_v/F_m		Performance Index	
	F	***p***	***F***	***p***	***F***	***p***
2009 (d.f. 5)						
Apšuciems G vs. Apšuciems V	2.26	0.207	0.142	0.725	2.76	0.172
Apšuciems G vs. Pope G	**13.20**	**0.022**	**9.54**	**0.037**	**18.90**	**0.012**
Apšuciems G vs. Pope V	0.89	0.399	**32.60**	**0.005**	**28.50**	**0.006**
Apšuciems V vs. Pope G	5.10	0.087	**10.50**	**0.032**	**26.60**	**0.007**
Apšuciems V vs. Pope V	0.69	0.452	**30.10**	**0.005**	**40.80**	**0.003**
Pope G vs. Pope V	**11.50**	**0.027**	0.38	0.573	0.08	0.795
2010 (d.f. 7)						
Apšuciems G vs. Apšuciems V	0.00	0.969	0.87	0.386	0.10	0.764
Apšuciems G vs. Pope G	0.00	0.973	0.09	0.771	2.10	0.197
Apšuciems G vs. Pope V	0.73	0.425	0.01	0.930	0.82	0.399
Apšuciems V vs. Pope G	0.00	0.987	5.03	0.066	**17.40**	**0.006**
Apšuciems V vs. Pope V	0.72	0.427	3.29	0.120	**6.04**	**0.049**
Pope G vs. Pope V	7.34	0.117	0.41	0.548	1.65	0.247
2011 (d.f. 7)						
Apšuciems G vs. Apšuciems V	0.01	0.950	1.910	0.217	2.80	0.145
Apšuciems G vs. Pope G	4.01	0.092	3.570	0.108	**6.04**	**0.048**
Apšuciems G vs. Pope V	0.04	0.852	2.360	0.175	4.92	0.068
Apšuciems V vs. Pope G	1.24	0.308	**14.30**	**0.009**	**27.2**	**0.002**
Apšuciems V vs. Pope V	0.01	0.938	**14.20**	**0.009**	**23.1**	**0.003**
Pope G vs. Pope V	2.02	0.205	0.28	0.613	0.07	0.797

Table S2. Results of ANOVA analysis of number of leaves and plant vitality index of *Saussurea esthonica* plants cultivated in substrate with different mineral nutrient composition. Degrees of freedom = 11.

**Soil Interactions**	**Number of Leaves**		**Vitality Index**	
	F	***p***	***F***	***p***
Control vs. "Apšuciems "	**23.13**	**0.001**	2.93	0.125
Control vs. "Pope"	3.10	0.116	4.72	0.062
Control vs. "Pope+"	**5.46**	**0.048**	**11.70**	**0.009**
"Apšuciems " vs. "Pope"	**14.86**	**0.005**	**8.60**	**0.019**
"Apšuciems " vs. "Pope+"	**23.12**	**0.001**	**16.65**	**0.004**
"Pope" vs. "Pope+"	0.24	0.636	0.17	0.688

Table S3. Results of ANOVA analysis of photosynthesis-related parameters of *Saussurea esthonica* plants cultivated in substrate with different mineral nutrient composition. Degrees of freedom = 25.

**Soil Interactions**	**Chlorophyll Content**		**Performance Index**		**F_v/F_m**		**RC/ABS**		**F_v/F_0**	
	F	***p***	***F***	***p***	***F***	***p***	***F***	***p***	***F***	***p***
Control vs. "Apšuciems "	**69.40**	**<0.001**	0.41	0.532	**5.76**	**0.026**	0.03	0.864	4.21	0.054
Control vs. "Pope"	**5.40**	**0.032**	0.84	0.371	4.21	0.054	0.01	0.134	4.62	0.094
Control vs. "Pope+"	**21.50**	**<0.001**	**8.64**	**0.008**	**7.93**	**0.011**	**6.71**	**0.018**	**11.20**	**0.003**
"Apšuciems " vs. "Pope"	**7.10**	**0.015**	2.04	0.169	**14.80**	**0.001**	0.00	0.975	**14.60**	**0.001**
"Apšuciems " vs. "Pope+"	0.77	0.389	**11.70**	**0.003**	**18.90**	**<0.001**	**6.85**	**0.017**	**23.40**	**<0.001**
"Pope" vs. "Pope+"	2.37	0.139	3.84	0.074	1.13	0.300	4.18	0.054	0.99	0.331

Table S4. Results of ANOVA analysis of number of leaves and plant vitality index of *Saussurea esthonica* plants cultivated in substrate with different water availability. Degrees of freedom = 11.

**Moisture Interactions**	**Number of Leaves**		**Vitality Index**	
	F	***p***	***F***	***p***
80% vs. 100%	**6.38**	**0.030**	2.09	0.179
80% vs. 150%	**16.70**	**0.027**	**21.40**	**0.001**
80% vs. 200%	**12.10**	**0.006**	**17.80**	**0.002**
100% vs. 150%	**10.70**	**0.009**	**16.00**	**0.003**
100% vs. 200%	**5.59**	**0.040**	**11.90**	**0.001**
150% vs. 200%	2.84	0.123	**5.19**	**0.046**

Table S5. Results of ANOVA analysis of photosynthesis-related parameters of *Saussurea esthonica* plants cultivated in substrate with different water availability. Degrees of freedom = 25.

**Moisture Interactions**	**Chlorophyll Content**		**Performance Index**		**F_v/F_m**		**RC/ABS**		**F_v/F_0**	
	F	***p***	***F***	***p***	***F***	***p***	***F***	***p***	***F***	***p***
80% vs. 100%	**23.30**	**<0.001**	**19.40**	**0.001**	**42.30**	**<0.001**	**27.20**	**<0.001**	**33.30**	**<0.001**
80% vs. 150%	**34.10**	**<0.001**	**16.10**	**0.001**	0.85	0.365	**31.90**	**<0.001**	0.77	0.390
80% vs. 200%	3.90	0.059	0.96	0.340	**22.40**	**<0.001**	2.10	0.160	**17.90**	**<0.001**
100% vs. 150%	0.20	0.660	**23.40**	**<0.001**	**29.30**	**<0.001**	0.06	0.818	**46.60**	**<0.001**
100% vs. 200%	**11.70**	**0.002**	**23.40**	**<0.001**	0.59	0.451	**19.80**	**<0.001**	0.50	0.485
150% vs. 200%	**19.00**	**0.002**	**25.30**	**<0.001**	**14.80**	**0.001**	**23.90**	**<0.001**	**25.30**	**<0.001**
